# Student-teacher relationship trajectories and mental health problems in young children

**DOI:** 10.1186/s40359-014-0027-2

**Published:** 2014-09-12

**Authors:** Lauren R Miller-Lewis, Alyssa CP Sawyer, Amelia K Searle, Murthy N Mittinty, Michael G Sawyer, John W Lynch

**Affiliations:** Discipline of Paediatrics, School of Paediatrics and Reproductive Health, University of Adelaide, Adelaide, South Australia 5005 Australia; Research and Evaluation Unit, Women’s and Children’s Hospital, Women’s and Children’s Health Network, 72 King William Road, North Adelaide, South Australia 5006 Australia; Discipline of Public Health, School of Population Health, University of Adelaide, Adelaide, South Australia 5005 Australia; Centre for Traumatic Stress Studies, School of Population Health, University of Adelaide, Adelaide, South Australia 5005 Australia; School of Social and Community Medicine, University of Bristol, Bristol, UK

**Keywords:** Early childhood, Mental health problems, Student-teacher relationship trajectories

## Abstract

**Background:**

This longitudinal study classified groups of children experiencing different trajectories of student-teacher relationship quality over the transition from preschool into school, and determined the strength of the association between different student-teacher relationship trajectories and childhood mental health problems in the second year of primary school.

**Methods:**

A community sample of 460 Australian children were assessed in preschool (age 4), the first school year (age 5), and second school year (age 6). Teachers at all three assessments reported on student-teacher relationship quality with the Student Teacher Relationship Scale. When the children were at preschool and in their second school year, parents and teachers rated children’s mental health problems using the Strengths and Difficulties Questionnaire.

**Results:**

Latent-class growth modelling identified two trajectories of student-teacher relationship quality: (1) a stable-high student-teacher relationship quality and (2) a moderate/declining student-teacher relationship quality trajectory. Generalised linear models found that after adjusting for family demographic characteristics, having a stable high quality student-teacher relationship trajectory was associated with fewer parent-rated and teacher-rated total mental health problems, and fewer conduct, hyperactivity, and peer problems, and greater prosocial behaviour at age 6. A stable high quality trajectory was also associated with fewer teacher-rated, but not parent-rated emotional symptoms. These effects remained after adjustment for levels of mental health problems at age 4.

**Conclusions:**

Findings suggest that early intervention and prevention strategies that focus on building stable high quality student-teacher relationships during preschool and children’s transition into formal schooling, may help reduce rates of childhood mental health problems during the early school years.

**Electronic supplementary material:**

The online version of this article (doi:10.1186/s40359-014-0027-2) contains supplementary material, which is available to authorized users.

## Background

Developmental contextual systems models hypothesise that children develop within the school and family contexts, and the key mechanisms through which they attain developmental competencies are the dyadic relationships they form with parents and teachers (Mashburn and Pianta 2006; Myers and Pianta 2008; O’Connor 2010). Teachers play an important role in the lives of children. Whilst parent relationships may be the most salient for very young children, the influence of teachers escalates with the increasing amounts of time young children spend in formal educational settings (Baker et al. 2008; Hamre and Pianta 2001; Silver et al. 2010). This is especially so in the early years of children’s education, when teachers often provide similar guidance and emotional support to that provided by parents (Hamre and Pianta 2001; Zhang and Sun 2011). The impact of children’s relationships with their teachers may be particularly salient at critical developmental periods (Silver et al. 2010). One such critical period is the transition from preschool into formal schooling, which is a time of new academic challenges in a more structured learning environment, involving complex changes in children’s roles, responsibilities, and relationships (Moore 2008; Silver et al. 2010). Caring and supportive student-teacher relationships may serve important functions for children’s successful adaptation to the school environment (Birch and Ladd 1997; Myers and Pianta 2008).

There is mounting evidence that high quality student-teacher relationships characterised by close supportive interactions and low conflict facilitate healthy child development (for reviews, see Mashburn and Pianta 2006; Myers and Pianta 2008; Roorda et al. 2011). Therefore, interventions that maximise the quality of relationships between teachers and children have the potential to improve children’s developmental outcomes (Myers and Pianta 2008). Intervening to improve young children’s mental health outcomes is particularly important because not only do approximately one in eight children experience significant mental health problems, mental health problems in the early years often persist into later childhood and adulthood, and are associated with adverse educational, psychosocial and health outcomes in adulthood (e.g., Caspi et al. 1996; Costello et al. 2005; Sawyer et al. 2000; Briggs-Gowan et al. 2006). Given the importance of early childhood development for later mental health, the transition to school period provides a unique opportunity to implement early interventions to young children at risk of developing mental health problems (Briggs-Gowan et al. 2006).

Although longitudinal studies of primary school children have found that poorer student-teacher relationships measured at a single point in time are associated with greater mental health problems in subsequent years (e.g., Meehan et al. 2003; Doumen et al. 2008; Pianta and Nimetz 1991; Buyse et al. 2009; Pianta et al. 1995; Tsai and Cheney 2012; Silver et al. 2005), only five longitudinal studies have examined student-teacher relationships and mental health problems during the transition from preschool into formal schooling (Howes 2000; Silver et al. 2010; Pianta and Stuhlman 2004; Peisner-Feinberg et al. 2001; Miller-Lewis et al. 2013). These studies reported that young children who had a poorer quality student-teacher relationship measured once during preschool were more likely to have greater subsequent mental health problems (Miller-Lewis et al. 2013), externalising behaviour problems (Silver et al. 2010; Pianta and Stuhlman 2004; Howes 2000; Peisner-Feinberg et al. 2001), internalising behaviour problems (Pianta and Stuhlman 2004), and poorer prosocial behaviour skills (Peisner-Feinberg et al. 2001; Howes 2000), by the time they were at primary school.

A number of potential mechanisms may link student-teacher relationships and mental health problems. Highlighting the bidirectional influences between an individual and their social context, there is theoretical and empirical indication that mental health problems and student-teacher relationships form part of a reciprocal transactional cycle, whereby children with early behaviour problems experience more barriers to forming positive relationships with teachers and subsequently experience more student-teacher conflicts, which in turn leads to more mental health problems, and so on (Doumen et al. 2008; Sameroff and MacKenzie 2003; Zhang and Sun 2011). There is also evidence that children seem to internalise negative interaction messages from teachers into their sense of self, with child self-esteem acting as an intervening mechanism linking student-teacher conflict with subsequent behaviour problems (Doumen et al. 2011). For example, a young child with emerging mental health problems (such as being withdrawn and shy or requiring regular behaviour management for misbehaviour) may be more challenging for a teacher to connect and engage with in a positive way. This lack of a positive connection, exhibited through less closeness and more conflict between the teacher and the child, may lead to diminishing self-esteem in the child, which in turn exacerbates mental health problems.

A major limitation of much of the previous research in this field is that the quality of student-teacher relationships was only assessed at a single point in time. It is important to examine the pattern of children’s student-teacher relationship quality over time as a measurement at a single point in time does not provide information about whether children’s relationship quality is improving, declining, or remaining stable over time. This is an important issue because there is evidence that children’s patterns of student-teacher relationship quality vary, particularly across the early school years (Jerome et al. 2009; Ladd and Burgess 1999; O’Connor and McCartney 2007). Indeed, O’Connor and McCartney (2007) identified distinct subgroups of children who followed moderate, inclining-high, and poor-declining trajectories of student-teacher relationship quality from preschool to third grade. Although the majority of children had moderate-to-good relationships with their teachers, they found a subgroup of children (13%) who had poor relationships throughout early school and experienced declining relationship quality over time. Further evidence from this research group has indicated that children with poor quality student-teacher relationships at school entry may follow a consistent pattern over time, where this relationship forms a basis for future relationships (O’Connor 2010).

Thus, given that children are exposed to multiple teachers and different educational settings during the transition from preschool into school, young children cannot be expected to all experience student-teacher relationships that change in the same direction across time. Theoretical understandings of developmental contextual systems and the transactional dyadic nature of the interactions within relationships between students and their teachers would instead suspect a multinomial heterogeneous pattern, in which both the strength and direction of change in relationship quality may vary between student-teacher dyads over time (Andruff et al. 2009; O’Connor 2010; Sameroff and MacKenzie 2003). Gaining knowledge about different trajectories of student-teacher relationship quality is important because repeated and cumulative experiences of relationship difficulties with teachers over time may have a greater and more lasting impact on children’s development than temporary or episodic difficulties experienced with a single teacher over a short period of time (Rudasill et al. 2010; Spilt et al. 2012a). It is particularly important to understand trajectories of student-teacher relationship quality in early childhood because the transition to formal schooling is a vulnerable period of child development characterised by major changes in children’s relationship dyads and changes into environments with different expectations of those relationships and child behaviour (Moore 2008; Silver et al. 2010; Green et al. 2008), all of which make stable student-teacher relationship quality somewhat less likely at this time (Green et al. 2008; Hughes and Cavell 1999; Spilt et al. 2012a). Therefore examining patterns and trajectories during the transition to school may provide useful insights into how student-teacher relationships and mental health are associated (Green et al. 2008).

Six longitudinal studies all conducted in the United States have found that healthy trajectories of student-teacher relationship quality are significantly associated with fewer externalising behaviour problems (O’Connor et al. 2011; O’Connor et al. 2012; Maldonado-Carreno and Votruba-Drzal 2011; Hughes and Cavell 1999; Ladd and Burgess 2001), fewer internalising behaviour problems (O’Connor et al. 2012; Maldonado-Carreno and Votruba-Drzal 2011; O’Connor et al. 2011), and better social skills (Berry and O’Connor 2010), in elementary school-aged children.

Only one study to date has examined children’s trajectories of student-teacher relationship quality and mental health problems during the period covering the transition into formal schooling. O’Connor et al. (2012) used longitudinal analyses to identify different trajectory groups based on (a) conflict and then (b) closeness in the teacher–child relationship from pre-kindergarten (age 4.5) to the fifth grade of school in the United States. They found that children in trajectory groups characterised by high or inclining levels of student-teacher conflict (16%) demonstrated higher levels of externalising behaviours in fifth grade (middle-childhood) than those in the low-conflict group. They also found that children in the stable-low-closeness trajectory group (3%) had higher levels of internalizing behaviours in fifth grade than those in the high-closeness student-teacher relationship group. Thus, while only a small proportion of children experienced these poorer quality student-teacher relationship trajectories, they showed substantially worse mental health outcomes. These associations remained after adjusting for the influence of pre-existing early childhood internalising and externalising behaviours, which led O’Connor et al. (2012) to conclude that children’s relationships with teachers may be able to change externalising and internalising behaviours developed in early childhood. However, it is not known whether the effects found by O’Connor et al. (2012) would be similar when examining mental health outcomes earlier in childhood (e.g., second year of school), or when investigating more specific aspects of mental health outcomes, such as hyperactivity, conduct problems, peer problems, emotional symptoms, or prosocial behaviour. The study by O’Connor et al. (2012) is also limited by examining mental health outcomes based on only parent-ratings of behaviour in the home setting, and by only adjusting for a limited range of likely confounding influences (gender, family income-to-needs, and maternal attachment). Such issues highlight avenues for further research to extend upon this important first study by O’Connor et al. (2012) to examine children’s trajectories of student-teacher relationship quality and mental health during the transition into formal schooling.

Another important factor is that it is not known whether the findings from the O’Connor et al. (2012) study would translate to other samples or cultures, such as Australia. All the previous studies examining student-teacher relationship trajectories and mental health outcomes in school children were conducted in the United States. Furthermore, we are aware of only four studies that have examined the association between student-teacher relationships and psychosocial outcomes in Australian children (Harrison et al. 2007; Miller-Lewis et al. 2013; Runions and Shaw 2013; Searle et al. 2013). Conducting research in other countries such as Australia is important because factors relevant to child developmental outcomes may be context and culture specific (Howard et al. 1999; Ungar 2012; Emerson and Einfeld 2010). The universal provision of one year of government-funded preschool for all 4–5 year old children in Australia, along with different distributions of socio-economic disadvantage, greater income mobility, and less spatial concentration of public housing, make it difficult to know how directly applicable findings from the United States would be to Australian children (Howard et al. 1999; Ungar 2012; Emerson and Einfeld 2010; Miller-Lewis et al. 2013). Additionally, Australian education legislation differs from many OECD countries because children can start formal schooling from the age of 4 and a half, and must be enrolled by age 6. The average age that Australian children start formal schooling is 5.2 years, with 75% starting school at age 5 or younger. This compares to an OECD average of 6.1 years, with only 10% starting school at age 5 or younger (OECD 2013). It is possible that the younger school commencement age of Australian children may increase the impact of student-teacher relationship quality on the wellbeing of these younger children. Furthermore, in the majority of cases, Australian preschools are stand-alone entities located separately from primary schools, staffed by dedicated early childhood preschool teachers who do not teach in schools. This means that the transition to formal schooling usually entails a change in physical location, along with changes in teachers, classmates, and teaching styles. Once at school, it is the norm to have a new teacher for each year of formal schooling thereafter. These factors may have important influences on student-teacher relationship trajectories experienced by Australian children.

### The present study

The aims of the present study were to (a) describe student-teacher relationship trajectories experienced by young Australian children between preschool (age 4), the first year (age 5) and the second year (age 6) of formal schooling; and (b) examine the role of these student-teacher relationship trajectories as predictors of mental health problems in children at age 6.

The present study extends the prior work of O’Connor et al. (2012) in several important ways. First, we examine specific subtypes of mental health outcomes in addition to overall levels of mental health problems. Second, we focus on these mental health outcomes during the early years of education. Third, we examine mental health outcomes reported by teachers in the school setting in addition to those reported by parents in the home setting. Fourth, we adjust for the potentially confounding effects of a more comprehensive set of child and family factors empirically shown to be associated with both student-teacher relationships and child mental health problems (e.g., gender, parental education and employment, welfare receipt, single parent family, parental psychological distress, and parenting styles (Beardslee et al. 1998; Bradley and Corwyn 2002; Fergusson and Horwood 2001; O’Connor et al. 2012; Spilt et al. 2012a; Miller-Lewis et al. 2014; Amato 2001)). Finally, according to Sabol and Pianta (2012), the present study also represents a needed extension upon existing research by being one of only a handful of studies on student-teacher relationships to be conducted outside of the United States.

Given our interest in examining different trajectories of student-teacher relationship quality, this study used methodology assessing individual variation in the trajectories of student-teacher relationships over time that are capable of detecting subgroups of children that follow different trajectories, rather than approaches that assess the average trajectories of relationship quality over time for the whole sample that can hide important differences between children (O’Connor and McCartney 2007). Similar to findings from previous studies of this kind (e.g., O’Connor and McCartney 2007; O’Connor et al. 2011; O’Connor et al. 2012) we hypothesised that there would be distinct subgroups of children who would experience different trajectories of student-teacher relationship quality over these early childhood years. In light of findings from a study of similarly aged children (O’Connor and McCartney 2007), we expected the majority of children would have moderate-to-good quality relationships with each of their teachers over time. Furthermore, we were interested to assess whether there were distinct trajectory subgroups of children experiencing poor quality relationships across the first years of school, who do not experience an improvement (or experience a decline) in relationship quality as they transition across different teachers.

Given that an enduring pattern occurring repeatedly over broader time intervals (i.e., poor relationships with multiple teachers over these school years) is believed likely to have a more enduring negative impact on a child’s development than processes that are episodic or inconsistent (i.e., one poor relationship in one school year) (Spilt et al. 2012a), we hypothesised that children with poorer-quality relationship trajectories with their teachers throughout their early years of education would have more mental health problems by the second school year, as compared to children with higher-quality student-teacher relationship trajectories. Specifically, children with stable experiences of high-quality student-teacher relationships were expected to have the lowest level of mental health problems, and any children experiencing stable poor-quality student-teacher relationships were expected to have the highest level of mental health problems, with the mental health of children experiencing pathways of change (i.e., inclining, declining, or quadratic trajectories of student-teacher relationship quality) falling in between these two more extreme trajectories. We expected the association between trajectories of student-teacher relationship quality and mental health outcomes would remain after adjusting for potential confounding factors, and that student-teacher relationship trajectories would influence multiple aspects of child mental health outcomes, including conduct, hyperactivity, peer, and emotional problems, as well as prosocial behaviour.

## Method

### Participants

This multi-informant longitudinal study focussed on 460 children who were part of a larger study on child development. Initially, 700 children attending the 27 government-funded preschools in one South Australian Government school district participated at baseline through the completion of a teacher-rated assessment. With 967 eligible children in the population, this represented a baseline response rate of 72.4%. The participating district is quite diverse, encompassing rural and remote areas as well as suburban areas, with some of these ranked at the greatest levels of socio-economic disadvantage in Australia. The demographic characteristics of this district overall resemble those for South Australia as a whole (Australian Bureau of Statistics 2004). At baseline, 51% of participating children were female, with a mean age of 4.7 years (*SD* = 0.3; modal age of 4 years).

In 2006, participation was sought from families of all children attending preschool in the district, a government-funded education programme which is available for up to 15 hours per week to all 4–5 year-old children for the year prior to beginning formal schooling. While attending preschool in South Australia is not compulsory, approximately 93% of eligible four year olds attend government-funded state preschools (Steering Committee for the Review of Government Service Provision 2008). At baseline, *both* a parent survey and a teacher survey were completed for 601 children (representing 62% of all preschool children in the district who were eligible for the study). Based on school district records, the participating children were of similar age and gender distribution to all preschool children in the district, but the percentage of children of Aboriginal/Torres Strait Islander (ATSI) descent was lower in the participating sample (1.4% versus 3.9%).

Two follow-up assessments were conducted after the children had commenced formal schooling. Assessment 2 occurred 12 months after the preschool assessment, when children were in their first year of formal schooling and had a mean age of 5.6 years (*SD* = 0.3). Assessment 3 occurred 24 months after the preschool assessment, when the children were in their second year of school and had a mean age of 6.7 years (*SD* = 0.4). For ease of presentation in the Results section, these assessments will be referred to as the Age 4, Age 5, and Age 6 assessments, respectively. All three teacher-rated assessments were completed for 642 children (retention rate = 92%), and full data on student-teacher relationship quality at all three assessments was available for 636 of these children (therefore analyses identifying student-teacher relationship trajectories utilised this sample of 636 children). Due to the structure of early education in Australia, it was not possible for the same teacher to complete the teacher-rated assessments over the three years of the study. Overall, 80% of participants in our study had a three different teachers complete the three assessments. In the majority of cases, the parent-reported surveys were completed by mothers (e.g., 92% at Assessment 1 and 3). For Assessment 3, when the children were in their second year of formal schooling, *both* the parent and teacher surveys were completed for 485 of the 601 children with parent- and teacher-reported baseline data (retention rate = 81%). The 116 children lost from the sample between Assessments 1 and 3 tended to be from families with more socio-economic disadvantage, and children with greater mental health problems (for further information, see Miller-Lewis et al. 2013). Of the original 700 participants recruited at age 4 for the first assessment, 485 participants had data available on the two mental health outcome variables at age 6 years needed for this study. Of these 485 participants, 460 participants had complete data on all of the study exposures, covariates, and outcomes. There is a lack of clear guidance from the methodological literature on the optimal approach to follow in regard to imputing outcome variable data. We decided to follow the more conservative approach by following von Hippel’s (von Hippel 2007) recommendations and so we did not impute outcomes where these were not observed. As such, we made the decision to not conduct missing data imputation, because the 25 extra participants (5%) that would be gained was highly unlikely to change the direction or size or significance of the effects observed in the present study, or the conclusions drawn. Table 1 provides demographic information and descriptive statistics for these 460 children with complete case data, as well as for the response sample for each study variable. Compared to the response sample, the sample with complete data were slightly less likely to have single parents, to be receiving government welfare, and to have unemployed parents. However in terms of the main variables of interest, student-teacher relationship quality and Strengths and Difficulties Questionnaire (SDQ) total difficulties scores, there was little difference in means between the response and complete case samples. Therefore the complete case analysis is unlikely to be biased.

**Table 1:** **Descriptive statistics on predictor and outcome variables for the response sample and the complete case sample**

	***Response sample***		***Complete case sample (n =460)***
	***n***	***M (SD)***	***M (SD)***
		***or %, range***	***or %, range***
*SDQ total difficulties score, (possible range 0–40)*			
*Parent-rated*			
Age 4	601	8.8 (4.9), 0-25	8.3 (4.6), 0-24
Age 6	516	8.3 (5.4), 0-30	8.2 (5.4), 0-30
*Teacher-rated*			
Age 4	700	5.8 (5.4), 0-30	5.2 (5.1), 0.25
Age 6	649	7.6 (6.7), 0-35	6.9 (6.2), 0-30
*Student-teacher relationship score, (possible range 15–75)*			
Age 4	700	67.2 (8.5), 28-75	68.2 (7.8), 31-75
Age 5	671	66.6 (8.8), 20-75	67.5 (7.9), 20-75
Age 6	645	65.9 (9.2), 33-75	66.8 (8.5), 36-75
*Child characteristics*			
Gender (female)	700	51.1%	51.5%
Age at preschool (baseline)	700	55.9 (3.9), 39-71	55.8 (3.8), 43-68
*Family characteristics during preschool (Age 4)*			
Parental warmth (main caregiver)	600	48.1 (4.4), 28-55	48.1 (4.2), 32-55
GHQ psychological distress (main caregiver)	595	1.5 (2.7), 0-12	1.4 (2.5), 0-12
Both parents unemployed^a^	601	10.3%	7.6%
Single parent family	600	16.5%	11.7%
Receiving government benefit	601	44.7%	39.8%
Mother’s education:	593	-	-
Less than year 12	-	27.5%	23.9%
Completed year 12	-	22.3%	21.7%
TAFE/Trade	-	32.7%	33.9%
Tertiary	-	17.5%	20.4%
Father’s education:	593	-	-
Less than year 12	-	32.1%	30.0%
Completed year 12	-	14.9%	15.9%
TAFE/Trade	-	43.0%	43.3%
Tertiary	-	10.0%	10.9%

^a^Both parents unemployed or an unemployed single parent.

### Measures

#### Child’s mental health problems

At age 4 and age 6, each child’s primary caregiving parent and their current teacher completed the Strengths and Difficulties Questionnaire (SDQ, Australian version for 4 to 10 year old children) (Goodman 1997), which is a psychiatric screening questionnaire designed to assess behaviour and emotions in children. The SDQ consists of 25 items divided between five subscales: Emotional Symptoms; Conduct Problems; Hyperactivity; Peer Problems; and Prosocial Behaviour. Respondents provide answers about the child’s behaviour (e.g., “often loses temper”) over the previous six months or the current school year, using a three-category response format of “not true”, “somewhat true”, or “certainly true”. Scores on each subscale can range from 0 to 10. A Total Difficulties score is generated by summing the subscale scores, with the exception of the prosocial subscale. Scores on Total Difficulties can range from 0 to 40, with higher scores indicating greater mental health problems. The SDQ has well-established psychometric properties, including strong relationships with diagnostic interviews (Goodman 2001; Stone et al. 2010; Warnick et al. 2008). In the present study, good internal consistency was evident with Cronbach’s Alphas of .79 and .86 on parent- and teacher-rated scores at age 4, and .83 and .88 on parent- and teacher-rated scores at age 6. The parent-rated SDQ subscales of emotional symptoms, peer problems, conduct problems, hyperactivity, and prosocial behaviour had Cronbach’s Alphas of .74, .62, .67, .80, and .66, respectively, and for the teacher-rated SDQ subscales, Cronbach’s Alphas were .81, .67, .82, .90, and .87, respectively. The internal consistency of the SDQ subscales in the present study was similar but predominantly better than that found in normative studies of the SDQ’s psychometric properties (Goodman 2001).

#### Student-teacher relationship quality

The main teacher of each child at the time of the three assessments was asked to describe the quality of their relationship with the child using the Short Form of the Student-Teacher Relationship Scale (STRS-SF), as used in the NICHD Study of Early Child Care and Youth Development (NICHD Early Child Care Research Network 2000; Pianta 2001). Teachers rate their perceptions of their relationship with the child on 15 items using a 5-point Likert scale (“definitely does not apply” to “definitely applies”). Items assess the level of warmth, closeness, and conflict in the relationship (e.g., “If upset, this child will seek comfort from me”). A total score is created from the sum of the 15 items, with higher scores reflecting a better quality relationship between the child and teacher. The STRS has good psychometric properties, including moderate correlations with behavioural ratings of teacher-child interaction (Pianta 2001; NICHD Early Child Care Research Network 2004b; NICHD Early Child Care Research Network 2004a). In the present study, excellent internal consistency was evident on the STRS-SF, with Cronbach’s Alphas of .90, .90, and .91 at Assessments 1, 2, and 3, respectively.

To ensure that the STRS measure functioned equivalently at the three different time-points for the different teachers who completed it, measurement invariance tests were conducted using Structural Equation Modelling in AMOS software (detailed results can be provided on request). Results of measurement invariance tests suggested the STRS functioned equivalently (and thus, teachers responded to the STRS items similarly) across time. Using nested models in structural equation modelling, a baseline unconstrained model where all parameters were freely estimated fit the data reasonably well, *χ*^*2*^ (231) = 987.43, *p* < .05; *CFI* = .95; *TLI* = .93; *RMSEA* = .04. Then, the negligible change in goodness-of-fit indices when constraining factor loadings to be invariant across time (*∆CFI* = .007, *∆TLI* = .001, *∆RMSEA* = .001) supported full metric invariance. Finally, in the last nested model, the minimal change in goodness-of-fit (*∆CFI* = .003, *∆TLI* = .001, *∆RMSEA* = .001) when most intercepts were constrained to be invariant supported partial scalar invariance. Thus, the STRS measurement model met the assumptions needed for comparing children’s STRS scores across time (Steenkamp and Baumgartner 1998; Van De Schoot et al. 2013), because these results suggest the STRS was being interpreted and completed similarly by teachers in preschool, the first, and second years of school.

#### Child’s family background

At the baseline assessment, the primary-caregiving parent provided information on five family socio-economic and demographic background characteristics. Whether the child was living in a single-parent versus a two-parent family was determined from the parent’s report on which parental figures currently live with the child. Responses to questions on the employment status of both the mother and the father (where present) were used to determine whether both parents were unemployed or not (or where a single parent-family, that parent was unemployed). The parent was also asked to indicate whether or not the family received any means-tested government welfare benefits for lower-income families. The responding parent also reported on the mother’s and the father’s highest level of completed educational qualifications.

The 12-item version of the widely-used General Health Questionnaire (GHQ-12) (Goldberg and Williams 1991) was utilised to assess psychological distress and impairment in the primary-caregiving parent. The standard binary scoring method (items scored as 0-0-1-1) was used, from which total scores can range from 0 to 12, with higher scores indicative of greater psychological distress (Donath 2001; Goldberg et al. 1997). The GHQ-12 has well-established psychometric properties, including detecting psychiatric cases (Goldberg et al. 1997; Donath 2001). The GHQ-12 Cronbach’s Alpha of .90 in the present study indicated good internal consistency.

Parental warmth was measured with the Warmth subscale of The Child-Rearing Practices Scale (Sanson 1996). The parent rated the frequency of expressing affection towards the child using a five-point Likert scale. The 11 items (e.g., “I often hug or hold my child for no particular reason”) are summed, with higher scores indicating greater parental warmth. The scale has adequate reliability, and is widely used in Australia (Australian Institute of Family Studies 2008; Sanson 1996). In the present study, the Cronbach’s Alpha of .86 demonstrated good internal consistency.

#### Data collection

With assistance from the research team, data collection for the baseline assessment was coordinated by the director at each preschool. Preschools distributed study information and consent forms, and teachers gave consenting parents the questionnaire. Parents returned completed surveys to the preschool in a sealed envelope. Teachers completed questionnaires on children once parent consent was obtained. For the second and third assessments, parent questionnaires were mailed directly to homes, and returned to the research team in pre-paid envelopes. Children were followed regardless of their school destination, and were attending over 100 different schools throughout the follow-up period. Nonetheless, the majority (69%) of children were attending government schools in the same district they attended preschool. A nominated liaison person at each school helped the research team distribute and collect the second and third teacher-rated assessments. At each assessment, parent-rated and teacher-rated surveys took approximately 30 and 10 minutes to complete, respectively. In order to allow time to get to know any newly commencing students, teachers were required to have interacted with them for a minimum of 5 weeks before providing their ratings about the child. Although teachers were required to wait a minimum of 5 weeks to complete surveys on new students, the vast majority of teachers had known the participating children for much longer. On average, the preschool teachers had been interacting with the children at preschool for 8 months (*SD =* 3.5). Only *n* = 2 (0.3%) preschool children had known their preschool teacher for less than 8 weeks. By the second assessment, 80.3% had been in their school class for more than one school term (i.e., more than 10 weeks), with a mean of 2.6 terms (*SD* = 1.1). By the third assessment, 99.2% of children had been at their school for more than one term, with a mean of 6.2 terms (*SD* = 1.4). Overall, 57 different teachers completed the preschool (age 4) assessments, 147 teachers completed the age 5 assessments, and 179 teachers completed the age 6 assessments. There was considerable variation in the number of children that school teachers completed surveys on (with a range of 1–20 [average = 5] at assessment 2 and 1–16 [average = 4] at assessment 3). The study methodology was approved by the Research Ethics Committees at the Women’s and Children’s Hospital Adelaide, and the South Australian Department of Education.

#### Statistical analyses

Statistical analyses were conducted using a two-stage process: (1) the creation of a latent exposure trajectory variable, and (2) assessing its association with our outcome variables of interest, i.e., mental health problems. The first stage involved using ratings on student-teacher relationships at ages 4, 5, and 6 to create a latent exposure variable representing distinct trajectories of student-teacher relationship quality over this period of time. A trajectory describes the developmental course of behaviour over age or time, and may be deflected by external events (Jones et al. 2001). We used a semi-parametric group-based modelling approach (Latent Class Growth Modelling), to estimate individual growth curves for each child, and then identify whether there were distinct subgroups of children who followed a distinct pattern of change over time (i.e., distinct linear and/or quadratic trajectories) (Nagin 1999; Andruff et al. 2009). Latent Class Growth Modelling was deemed the most suitable analysis strategy (over and above standard growth trajectory analyses of averaged intercept and slope), because a homogeneous pattern (e.g., where all participants are expected to change in the same direction over time, with only the degree of change varying between them) was very unlikely when in most cases there were three different student-teacher dyads over the course of the study. A multinomial heterogeneous pattern was expected, because it was possible that distinct subgroups children would experience improving student-teacher relationship trajectories over time, while other subgroups experienced declining relationship trajectories, or an improvement with their second teacher and then a decline with their third teacher (i.e., a quadratic trajectory), and other groups whose relationships with teachers over time remained stable (Andruff et al. 2009; O’Connor and McCartney 2007; O’Connor et al. 2011; O’Connor et al. 2012).

The Latent Class Growth Modelling was performed using the PROC TRAJ macro in SAS (Jones and Nagin 2007). In order to provide the best estimate of trajectories of student-teacher relationship quality, trajectories were estimated using the full sample that had complete relationship quality scores at the three time points of interest (*n* = 636). To identify the best fitting number of trajectories, we fit a series of models and utilised a number of criteria including: (1) whether the trajectory shape was statistically significant at a *p* level of .05; (2) whether the Bayesian Information Criteria (BIC) value for models with increasing numbers of groups showed evidence of improved fit; (3) whether at least 5% of the sample were identified as following each trajectory (given that we were not using a clinical sample, it was unlikely that we would identify valid trajectories that are only followed by a small fraction of the population), and; (4) whether the probability of group membership for each trajectory was 0.70 or higher, indicating that the identified trajectories were indeed grouping together individuals with similar patterns of change over time (Andruff et al. 2009; Nagin 1999). As recommended by Nagin and Odgers (2010), the number of groups in the final model chosen was based on a combination of these criteria.

In PROC TRAJ, the uncertainty in latent class membership is accounted for by the latent class variable C being treated as missing data problem and a joint likelihood is estimated using the outcome, the covariate, and the latent variable C. The estimation of parameters is then done by using expectation maximization (EM) algorithm on the complete log likelihood function of outcome, exposure, and the latent class variable C. Use of EM algorithm allows for addressing the uncertainty in the latent class membership (Roeder et al. 1999). In other words, using Bayesian methods of estimation, the STRS latent class trajectory variable is treated as an observed variable with completely missing data. In doing this, the expectation maximisation algorithm (a missing data method) computes values of the STRS trajectory score from the joint distribution of the STRS scores at all three time points, along with the modelled STRS trajectory scores, using iterative processes.

The second stage of statistical analyses examined the strength of the association between the latent student-teacher relationship trajectory groups and parent-rated and teacher-rated SDQ total difficulties and five subscale scores in the second year of school using the sample with complete information on the outcome and the confounding variables of interest (*n* = 460). We examined these associations using Generalized Linear Models in Stata version 12.1 (Stata Corp, College Station, TX, USA). In multivariate analyses, we adjusted for several potential confounders of the association between children’s student-teacher relationship trajectories and children’s mental health scores in the second school year. These confounders were identified on the basis of evidence from previous studies describing factors that may influence children’s relationship with their teachers and their mental health problems (e.g., Amato 2001; Beardslee et al. 1998; Bradley and Corwyn 2002; Fergusson and Horwood 2001; Howes 2000; O’Connor and McCartney 2006, 2007; O’Connor et al. 2012; Jerome et al. 2009; Spilt et al. 2012a; Ladd et al. 1999; Pianta and Stuhlman 2004; Miller-Lewis et al. 2013; Miller-Lewis et al. 2014), and *a priori* on the basis of Directed Acyclic Graphs, which are visual diagrams summarising assumptions regarding potential causal links between variables (Greenland et al. 1999). In the final analysis models we also adjusted for the child’s corresponding SDQ scores (informant-specific) at age 4. Therefore, the final models represented a test of the association between student-teacher relationship trajectories and the *change* in SDQ mental health scores between preschool and the second school year, rather than the absolute level of SDQ mental health in the second school year. When adjusting for baseline assessments of the outcome, it is possible that under certain conditions this can introduce bias due to associated measurement error (Glymour et al. 2005). For example, this can occur when measurement error (or rater bias) from parent-rated scores at baseline are associated with measurement error for parent-rated outcome scores. When this occurs, it introduces bias into the analysis and may result in a spurious association. Therefore, these final models should be interpreted with some caution. We included the model adjusting for the SDQ score at preschool as a final strong test of the association between student-teacher relationship quality trajectories and SDQ scores in the second year of school, as SDQ scores at age 4 are likely to reflect, in part, information on a number of potentially confounding factors which we were not able to adjust for in our analysis.

Our data was clustered at the preschool level because participants were recruited from 27 preschools. Generally the preferred analysis for school clustered data is to conduct multilevel analysis (Goldstein 1987). We calculated intra-class correlations (ICCs) for the outcome variables in STATA. The ICCs for age 6 parent-rated and teacher-rated SDQ total difficulties were not significant (0.02, 95% CI 0.00-0.06 and 0.06, 95% CI 0.00-0.12, respectively), indicating small cluster effects and only modest homogeneity within the clusters. Additionally, the largest design effect (calculated by: 1+ (ICC*[average cluster n-1]) was 1.96 (based on the average of 17.03 children per preschool in the study sample), which is a design effect within the range expected for a well-designed study (United Nations Department of Economic and Social Affairs Statistics Division 2001; Shackman 2001). This indicates that treating the cluster sample elements as though they had been selected by a simple random sample and analysing them would give the same results (Groves et al. 2009). Hence we chose to analyse them using simple generalised linear regression models.

## Results

### Trajectories of student-teacher relationship quality

Table 1 shows the scores for student-teacher relationship quality at the ages 4, 5, and 6 assessments. The scores on the Student-Teacher Relationship Scale were positively skewed at each of the assessment time points, with the majority of teachers reporting moderate-to-good quality relationships with their students (inter-quartile ranges at age 4, age 5, and age 6 were 41–75, 36–75, and 38–75 respectively). On average for the whole sample, student-teacher relationship quality scores decreased over time (see Table 1). Moderate positive correlations were found between the scores on student-teacher relationship quality across the three assessments. Age 4 scores correlated with age 5 scores at *r* = 0.41, *p* < .001; age 4 scores correlated with age 6 scores at *r* = 0.27, *p* < .001; and age 5 scores correlated with age 6 scores at *r* = 0.49, *p* < .001.

**Figure 1:**
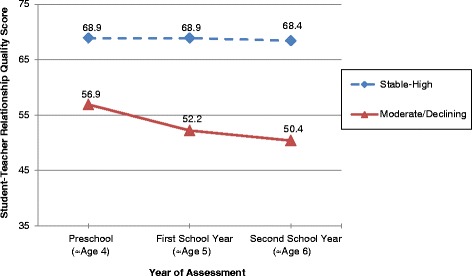
**Observed trajectories of student-teacher relationship quality from preschool through to the second school year.**

Latent Class Growth Modelling identified two trajectories of student-teacher relationship quality: (1) ‘Stable-High Relationship Quality’ (*n* = 550, 86.5% of the sample), and (2) ‘Moderate/Declining Relationship Quality’ (*n* = 86, 13.5% of the sample) (see Figure 1). Student-teacher relationship trajectories were found to be linear in shape across the three time points. No significant quadratic trajectories were detected. The BIC value for one linear trajectory was −6244, and for two linear trajectories the BIC value was −6103.03, indicating improved fit. We calculated the estimate of the log Bayes factor in order to further examine this evidence of improved fit (Jones et al. 2001). The estimate of the log Bayes factor was 276.5 suggesting strong evidence for improved model fit (Jones et al. 2001). There was no evidence for a three trajectory model because the third trajectory was not found to be statistically significant (*p* = .55) and the third trajectory included less than 5% of the sample (2.2%).

Finally, for the two trajectory model, the probability of correct classification into group membership for trajectory 1 was 0.89, while for trajectory 2 was 0.98, again indicating that the two identified trajectories successfully grouped individuals with similar patterns of change over time. The two trajectory model identified one statistically significant trajectory (*p* < .001) and a second trajectory that approached statistical significance (*p* = .08). Given the strong evidence of good model fit and the presence of two distinct trajectories, the two trajectory model was selected as the final model.

Table 2 shows the mean relationship quality scores and 95% confidence intervals (CI) at each of the three time points for the two trajectory groups identified. The moderate/declining trajectory had moderate levels of student-teacher relationship quality at age 4, which then declined at age 5, and declined again at age 6. The stable high trajectory had high quality student-teacher relationships at ages 4, 5, and 6, that remained unchanged over the three assessments.

**Table 2:** **Scores at each assessment on student-teacher relationship quality and SDQ total difficulties for the identified relationship quality trajectory groups**

	***Moderate/declining***			***Stable-high***		
	***Student-teacher relationship trajectory***			***Student-teacher relationship trajectory***		
	***n***	***M (SD)***	***95% CI***	***n***	***M (SD)***	***95% CI***
*Relationship quality scores*						
Age 4	86	56.9 (11.9)	54.4 – 59.5	550	68.9 (6.3)	68.4 – 69.5
Age 5	86	52.2 (10.1)	50.1 – 54.4	550	68.9 (5.9)	68.4 – 69.4
Age 6	86	50.4 (9.5)	48.3 – 52.4	550	68.4 (6.3)	67.9 – 68.9
*Parent-rated SDQ total scores*						
Age 4	47	10.1 (0.6)	8.8 – 11.3	413	8.1 (0.2)	7.6 – 8.5
Age 6	47	12.1 (0.9)	10.3 – 13.9	413	7.8 (0.2)	7.2 – 8.2
*Teacher-rated SDQ total scores*						
Age 4	47	11.7 (1.0)	9.7 – 13.6	413	4.4 (0.2)	4.0 – 4.8
Age 6	47	16.9 (0.9)	15.0 – 18.7	413	5.7 (0.2)	5.2 – 6.2

### Associations between student-teacher relationship quality trajectories and mental health at age 6

The lower section of Table 2 displays the mean scores and 95% CIs on SDQ total mental health problems at age 4 and 6 years for the two student-teacher trajectory groups. On both parent- and teacher-rated SDQ total scores at age 4 and 6 years, children in the stable-high student-teacher relationship trajectory group had lower mean scores on SDQ total difficulties than children with a moderate-declining trajectory. This difference between groups was more pronounced by age 6, and when SDQ scores were reported by teachers rather than parents. For example, by age 6, children with stable-high relationship trajectories had a teacher-rated SDQ difficulties mean score of 5.7 (well within the normal range), whereas children in the moderate-declining trajectory group had a mean SDQ score of 16.9, which is above the abnormal/clinical cut-off for teacher-rated SDQ scores. Consistent with results in Table 2, Table 3 shows that higher scores on student-teacher relationship quality at age 4, age 5, and age 6 were each significantly associated with lower SDQ total difficulties scores at age 6 rated by parents and teachers.

**Table 3:** **Associations between teacher-rated student-teacher relationship quality scores at age 4, age 5 and age 6, and SDQ total difficulties scores at age 6 as rated by parents and teachers (n = 460)**

	***Parent-rated SDQ total score age 6***			***Teacher-rated SDQ total score age 6***		
	***B*** ^***a***^	***95% CI***	***p***	***B*** ^***a***^	***95% CI***	***p***
*Relationship quality score*						
Age 4	−0.11	−0.18 - −0.05	<.001	−0.27	−0.34 - −0.19	<.001
Age 5	−0.15	−0.21 - −0.09	<.001	−0.32	−0.38 - −0.25	<.001
Age 6	−0.18	−0.24 - −0.13	<.001	−0.48	−0.53 - −0.43	<.001

^a^Unstandardised regression coefficient.

A series of Generalised Linear Models examining the association between age 6 SDQ total difficulties scores and student-teacher relationship trajectory groups are shown in Table 4. The results from Model 1 in Table 4 indicate that children with trajectories of stable-high student-teacher relationship quality had significantly lower parent-rated SDQ total difficulties scores at age 6 than children with a moderate/declining student-teacher relationship quality trajectory. On Teacher-rated SDQ total difficulties at age 6, children with a stable-high student-teacher relationship quality trajectory scored significantly lower on SDQ total difficulties than children with a moderate/declining trajectory. Table 4 indicates that the age 6 SDQ total difficulties scores for the stable-high quality student-teacher relationship group attenuated but remained statistically significant after adjustment for child and family background characteristics (Model 2), and after adjustment for the corresponding baseline SDQ total difficulties scores at age 4 (Model 3). (See Additional file 1 for the full set of regression coefficients from the adjusted models.) Based on the final adjusted model (Model 3), children displaying a stable-high student-teacher relationship quality trajectory had a parent-rated SDQ total difficulties score at age 6 that was 2.83 points lower than children with a moderate/declining student-teacher relationship quality trajectory. This was a medium sized effect (*Cohen’s d* = 0.53) according to Cohen’s (1988) criteria for interpreting effect sizes. On teacher-rated SDQ total difficulties at age 6, children with a stable-high student-teacher relationship quality trajectory scored 7.98 points lower than children with a moderate/declining student-teacher relationship quality trajectory (*Cohen’s d* = 1.4). This was a large effect size (Cohen 1988; Wilson n.d.).

**Table 4:** **Regression coefficients describing association between SDQ total difficulties scores at age 6 (Parent- and Teacher-rated) and student-teacher relationship quality trajectories (Stable High versus Moderate/Declining), n = 460**

	***Parent-rated SDQ total score at age 6***			***Teacher-rated SDQ total score at age 6***		
	***B***	***95% CI***	***p***	***B***	***95% CI***	***p***
*Model 1 (unadjusted)*						
Moderate/Declining	ref	-	-	-	-	-
Stable high	−4.38	−5.97 - −2.78	<.001	−11.15	−12.73 - −9.57	<.001
*Model 2 (adjusted)* ^*a*^						
Moderate/Declining	ref	-	-	-	-	-
Stable high	−3.69	−5.32 - −2.07	<.001	−10.15	−11.79 - −8.51	<.001
*Model 3 (Model 2 + baseline SDQ)* ^*b*^						
Moderate/Declining	ref	-	-	-	-	-
Stable high	−2.83	−4.11 - −1.54	<.001	−7.98	−9.69 - −6.28	<.001

^a^Adjusted for: child age at preschool, child gender, Parent distress, Parental warmth, maternal and paternal education and employment, receiving welfare benefit, and single parent family status. ^b^Adjusted for: child age at preschool, child gender, Parent distress, Parental warmth, maternal and paternal education and employment, receiving welfare benefit, and single parent family status, and the corresponding informant-specific SDQ Total difficulties score at age 4 (parent-rated and teacher-rated, respectively).

*Note.* The Moderate/Declining trajectory group was treated as the reference group in these analyses.

*Note.* See Additional file 1 for more detailed tables with the full set of coefficients for all variables included in the adjusted models (Models 2 and 3).

We then repeated these analyses examining the effect of children’s student-teacher relationship trajectories separately on each of the five subscale scores from the SDQ. Table 5 (Model 1) shows the regression coefficients describing the association between having a stable-high versus a moderate/declining relationship trajectory and (1) emotional difficulties, (2) peer problems, (3) hyperactivity, (4) conduct problems, and (5) prosocial skills reported by parents and teachers at age 6. The results indicate that children with stable-high student-teacher relationship quality trajectories had significantly lower parent- and teacher-rated peer problems, hyperactivity, and conduct problems, and higher prosocial skills, by age 6 years. For emotional difficulties, children with stable-high relationship quality trajectories had significantly lower emotional difficulties at age 6 as rated by teachers, but not when rated by parents. Table 5 (Models 2 and 3) show that the age 6 SDQ subscale scores for the stable-high student-teacher relationship quality group attenuated but remained statistically significant after adjustment for child and family background characteristics, and for the corresponding informant-specific baseline SDQ subscale score (parent-rated and teacher-rated, respectively).

**Table 5:** **Regression coefficients describing association between SDQ subscale scores at age 6 (Parent- and Teacher-rated) and student-teacher relationship quality trajectories (Stable High versus Moderate/Declining), n = 460**

	***Parent-rated SDQ subscale score at age 6***			***Teacher-rated SDQ subscale score at age 6***		
	***B***	***95% CI***	***p***	***B***	***95% CI***	***p***
*1. SDQ emotional difficulties subscale score at age 6*						
*Model 1*						
Moderate/declining	ref	-	-	-	-	-
Stable high	−0.02	−0.62 - 0.58	.942	−1.46	−2.06 - −0.85	<.001
*Model 2* ^*a*^						
Moderate/declining	ref	-	-	-	-	-
Stable high	−0.14	−0.75 - 0.47	.661	−1.52	−2.16 - −0.88	<.001
*Model 3* ^*b*^						
Moderate/declining	ref	-	-	-	-	-
Stable high	−0.14	−0.63 - 0.36	.592	−1.37	−2.01 - −0.73	<.001
*2. SDQ Peer Problems Subscale Score at Age 6*						
*Model 1*						
Moderate/declining	ref	-	-	-	-	-
Stable High	−0.59	−1.05 - −0.14	.011	−1.75	−2.25 - −1.25	<.001
*Model 2* ^*a*^						
Moderate/declining	ref	-	-	-	-	-
Stable high	−0.39	−0.86 - 0.08	.103	−1.56	−2.09 - −1.03	<.001
*Model 3* ^*b*^						
Moderate/declining	ref	-	-	-	-	-
Stable high	−0.47	−0.89 - −0.05	.030	−1.42	−1.95 - −0.89	<.001
*3. SDQ Hyperactivity Subscale Score at Age 6*						
*Model 1*						
Moderate/declining	ref	-	-	-	-	-
Stable high	−2.21	−2.91 - −1.51	<.001	−4.46	−5.25 - −3.68	<.001
*Model 2* ^*a*^						
Moderate/declining	ref	-	-	-	-	-
Stable High	−1.87	−2.60 - −1.14	<.001	−3.73	−4.53 - −2.94	<.001
*Model 3* ^*b*^						
Moderate/declining	ref	-	-	-	-	-
Stable high	−1.22	−1.81 - −0.62	<.001	−2.40	−3.18 - −1.63	<.001
*4. SDQ Conduct Problems Subscale Score at Age 6*						
*Model 1*						
Moderate/declining	ref	-	-	-	-	-
Stable high	−1.55	−2.02 - −1.08	<.001	−3.48	−3.90 - −3.06	<.001
*Model 2* ^*a*^						
Moderate/declining	ref	-	-	-	-	-
Stable high	−1.30	−1.77 - −0.82	<.001	−3.34	−3.79 - −2.89	<.001
*Model 3* ^*b*^						
Moderate/declining	ref	-	-	-	-	-
Stable high	−1.10	−1.51 - −0.69	<.001	−2.87	−3.35 - −2.38	<.001
*5. SDQ Prosocial Skills Subscale Score at Age 6*						
*Model 1*						
Moderate/declining	ref	-	-	-	-	-
Stable high	1.08	0.61 - 1.56	<.001	3.30	2.65 - 3.96	<.001
*Model 2* ^*a*^						
Moderate/declining	ref	-	-	-	-	-
Stable high	0.77	0.28 - 1.25	.002	2.83	2.15 - 3.50	<.001
*Model 3* ^*b*^						
Moderate/declining	ref	-	-	-	-	-
Stable high	0.60	0.16 - 1.03	.007	2.48	1.78 - 3.18	<.001

^a^Adjusted for: child age at preschool, child gender, Parent distress, Parental warmth, maternal and paternal education and employment, receiving welfare benefit, and single parent family status. ^b^Adjusted for: child age at preschool, child gender, Parent distress, Parental warmth, maternal and paternal education and employment, receiving welfare benefit, and single parent family status, and the corresponding informant-specific SDQ Subscale score at age 4 (parent-rated and teacher-rated, respectively).

*Note.* The Moderate/Declining Relationship trajectory group was treated as the reference group in these analyses.

## Discussion

This prospective longitudinal study expands the body of research examining trajectories in student-teacher relationship quality and their influence on young children’s mental health problems. Using latent class growth modelling, we identified two distinct subgroups of student-teacher relationship trajectories across the transition from preschool to school: a predominant stable-high student-teacher relationship quality trajectory, and a more atypical moderate/declining student-teacher relationship quality trajectory. The trajectories found here in our sample of Australian children were similar to those found by O’Connor and McCartney (2007), who also identified a group of 13% in their US sample that had poor-declining student-teacher relationships. Our results suggest that high quality student-teacher relationships appear to be normative, with children who start off in preschool with high quality relationships continuing on a stable high-quality trajectory. It is notable that despite most of the children having three different teachers for each of the three years of the study, the majority of children in the sample experienced stable high quality student-teacher relationship trajectories. Nonetheless, a small but significant percentage of young children experienced poorer quality student-teacher relationships in preschool, which got worse over time as children transitioned into formal school. Therefore, these early relationships in preschool seem to form a basis for future relationships (Spilt et al. 2012a; O’Connor 2010), suggesting that building high quality student-teacher relationship trajectories in school needs to start with establishing a strong student-teacher relationship in preschool.

In the present study, we found that trajectories of student-teacher relationship quality during the preschool-school transition were associated with children’s mental health problems in the second school year. Specifically, compared to children who experienced moderate student-teacher relationship quality in preschool which then declined as they entered formal schooling, children who experienced a stable high-quality relationship with their teachers throughout the transition from preschool to school had significantly lower levels of parent- and teacher-rated mental health problems in the second school year. This pattern was consistent for overall mental health problems, but also for specific types of problems, including conduct problems, hyperactivity, and peer problems. Consistent with one of the few studies to examine social skills (Berry and O’Connor 2010), we also found that a stable high-quality student-teacher relationship trajectory was associated with greater parent- and teacher-rated prosocial behaviour skills in the second school year.

The association between student-teacher relationship trajectories and later mental health outcomes remained significant after adjusting for a battery of child and family socio-demographic factors, suggesting that their associations were not a result of confounding or spurious associations that could be attributed to other variables known to be associated with both student-teacher relationships and mental health (O’Connor et al. 2011). Furthermore, these associations also remained after accounting for the effects of mental health problems reported in preschool. This indicates that not only were student-teacher relationship trajectories associated with mental health problems in the second school year, they were also predictive of changes in mental health problems over time between preschool and the second school year. This suggests that children’s cumulative experiences in relationships with their teachers may have the capacity to change the course of mental health problems developed in early childhood (O’Connor et al. 2012; Baker et al. 2008). Although some bias may have been introduced by adjusting for baseline levels of the SDQ outcome (as previously discussed (Glymour et al. 2005)), given the pattern of effect was consistent with results without adjustment for baseline SDQ scores, it is unlikely that substantial bias has been introduced as a result of this adjustment.

Our findings specifically examining emotional difficulties as a mental health outcome were less consistent than our findings on other mental health outcomes. We found that a stable high-quality student-teacher relationship trajectory was associated with lower levels of emotional difficulties in the second school year only when these difficulties were reported by teachers, and not by parents. Previous research has also found larger effects for teacher-rated internalising problems than for those rated by parents (Maldonado-Carreno and Votruba-Drzal 2011). It is possible that student-teacher relationship trajectories have greater influence on the presentation of emotional difficulties in the school environment than in the home environment - for example if a child has a poor relationship with their teacher it may affect a child’s behaviour in class more than at home. However, it is also possible that the association found between student-teacher relationship trajectories and emotional difficulties reported by teachers in the second school year may be at least in part explained by the shared-rater variance resulting from the second-school-year teacher rating both student-teacher relationships and emotional difficulties at this time.

This study has several strengths, including the use of multiple longitudinal assessments of student-teacher relationships during the important school transition period, multiple informants to assess mental health problems, and adjusting for an array of potentially confounding variables. However, the following limitations should be considered when interpreting the results. First, because the teacher at each assessment was the only informant on student-teacher relationship quality, it is unknown if the teachers’ perceptions biased their reports. Given that we were relying solely on teacher’s reports of student-teacher relationship quality, it is possible that these reports may be mostly a function of teacher perceptions, particularly for the small number of children and teachers who had known each other for less than two months. For this reason, our results need to be interpreted with some caution, especially for those child-teacher dyads who had not known each other for long. Obtaining additional reports on student-teacher relationships from the child or parent’s perspective and direct classroom observations would be worthwhile, and may result in a different pattern of findings (Harrison et al. 2007). Second, student-teacher relationship trajectories were more strongly associated with teacher reports of mental health problems than parent reports. It is possible that because mental health problems and the component of student-teacher relationship trajectories at the third assessment were reported by the same teacher, common-rater bias may have inflated these associations, even though the first and second assessments of student-teacher relationships were reported by different teachers. Third, our student-teacher relationship trajectories did not fully temporally precede our mental health outcomes, because the third assessment utilised to create our trajectory groups was measured contemporaneously with our mental health outcomes in the second school year. Our results can only suggest but not confirm possible causal sequences. Future research would benefit from conducting at least four assessments over time, and in particular concentrating multiple assessments around the critical point of transition from the completion of preschool into the onset of formal schooling. This may help illuminate potential reciprocal and transactional processes within student-teacher relationships and mental health over time (e.g., Doumen et al. 2008; Zhang and Sun 2011). Fourth, we lacked information on characteristics of teachers, classes, and schools (e.g., class size; teacher qualifications) that may have held associations with student-teacher relationship trajectories. Inclusion of this information in the adjusted models may have altered the pattern of results. In particular, we do not know exactly how many children were in each school classroom during the follow-up assessments. Whilst Australian regulations limit junior primary school class sizes to a maximum of 26 students, it is possible that variation in class size may influence the ability of teachers to form meaningful relationships with all their students. Fifth, not all children in each school classroom were assessed (only those who participated in the preschool survey were followed-up). Over 100 schools and over 180 different teachers were involved in the follow-up assessments, and there was large variation in the number of children that school teachers completed surveys on. This meant it was not feasible to determine whether clustering effects such as anchoring were occurring at the school classroom level for our sample at assessment 2 and 3. For instance, where a student was in a school class with low-ranked peers (i.e., children with lower baseline preschool levels of student-teacher relationship), this student might get a higher ranking due to the biased comparative assessment of the teacher. Finally, our findings may not be fully generalizable to the broader population of Australian preschool children. While the demographic characteristics of our cohort resembled the larger population of South Australian children, Aboriginal and Torres Strait Islander (ATSI) children were underrepresented in our cohort, and there was a higher rate of sample attrition for children from families experiencing more adverse socio-economic circumstances (Australian Bureau of Statistics 2004; Miller-Lewis et al. 2013).

## Conclusions

This study found that children with stable high student-teacher relationship quality trajectories across preschool, the first school year, and the second school year had fewer mental health problems in the second school year than children with moderate/declining quality trajectories. Student-teacher relationship trajectories remained associated with the level of mental health problems in the second school year, even after accounting for the effects of potentially confounding family socio-demographic characteristics and mental health problems in preschool. This suggests that children’s relationships with their teachers may have an important influence on the developmental course of childhood mental health problems. The results of the present study suggest that helping teachers and children develop stable high quality relationships may improve children’s mental health following school entry. Teachers are uniquely placed to provide children with the opportunity to experience a healthy relationship with a supportive adult, which is of fundamental importance to children’s development (Mashburn and Pianta 2006; Meehan et al. 2003; O’Connor et al. 2012; Ladd and Burgess 1999). This study suggests that high-quality stable student-teacher relationships across the preschool-to-school transition may represent a broad and far-reaching factor that protects against the development of a number of different mental health problems in children, including conduct problems, hyperactivity, peer problems, prosocial behaviour, and emotional difficulties in the school environment.

Therefore, intervention strategies that successfully foster stable high-quality student-teacher relationships during preschool and the transition into school, may be capable of reducing a broad range of mental health problems, not just one specific type of problem. Building stable high-quality student-teacher relationship trajectories may prove difficult during the transition into school, given the major changes that occur during this time. Organising a series of transition visits for the preschool children to visit their new school classroom to meet their teacher before commencing school may prove fruitful. But given we found children with poorer quality student-teacher relationship trajectories had considerably poorer relationships with their preschool teacher from the outset, it may be more fruitful to foster high quality student-teacher relationships in preschool and child care, before the advent of formal schooling, because this may form a strong basis for future stable high-quality student-teacher relationship trajectories at school, which may in turn reduce rates of mental health problems in young children.

Organisational strategies, teacher professional development, and early childhood interventions that increase the opportunity for children and teachers to experience healthy interactions and to establish and maintain high-quality relationships may help to reduce children’s mental health difficulties in the early years of school. It is important that early intervention strategies to improve student-teacher relationships consider both interaction partners (teacher and child), as strategies that combine teacher training with child intervention should exhibit the most success (Doumen et al. 2008; Zhang and Sun 2011). Intervention strategies for children may include social skills training and fostering self-worth. Strategies for teachers may include interventions such as ‘relationship-focussed reflection’ upon relationships with individual children, ‘banking time’ with children (one-on-one child-directed sessions to increase closeness), and behaviour management interventions such as the ‘Good behaviour game’ which focuses on rewarding positive behaviour in order to facilitate the greater frequency of positive interactions (as opposed to negative interactions) between students and teachers (Leflot et al. 2010; Driscoll et al. 2011; Spilt et al. 2012b; Doumen et al. 2011; Sabol and Pianta 2012). Given the important influence of student-teacher relationships on children’s mental health, teacher training and professional development should ensure teachers are fully aware of the impact they can have on children’s mental health (Doumen et al. 2011). Knowledge of risk factors associated with poor student-teacher relationship trajectories may also usefully suggest potential targets for interventions (Zhang and Sun 2011). Research with this sample (Miller-Lewis et al. 2014) and others (O’Connor 2010; O’Connor and McCartney 2007; O’Connor et al. 2012; Jerome et al. 2009) has found that these risk factors include being a boy, poorer social skills, significant mental health problems in preschool, exposure to poorer parenting, low maternal education, poor classroom management, and having a poorer quality relationship with kindergarten teachers. Knowledge on such risk factors for poor quality student-teacher relationship trajectories would be useful to impart in teacher training and professional development, as this knowledge may make teachers more mindful of counteracting this possibility by consciously adjusting the frequency and type of their interactions with children who could be considered at-risk for poorer quality relationships. Multifaceted early intervention strategies tackling student-teacher relationships through multiple avenues such as those described here are likely to have a stronger and more lasting impact on the long-term mental health and wellbeing of children, helping to place them on more positive developmental trajectories.

## Additional file

Additional file 1
**Full set of regression coefficients from adjusted models describing association between SDQ total difficulties scores at age 6 (Parent- and Teacher-rated) and student-teacher relationship quality trajectories (Stable High versus Moderate/Declining), n = 460.**
