# Supplementary material for: Student-teacher relationship trajectories and mental health problems in young children
Source: BMC Psychol. 2014 Sep 12;2(1):27. doi: 10.1186/s40359-014-0027-2 (PMC4317136; doi:10.1186/s40359-014-0027-2)
Supplement: Additional file 1 — Full set of regression coefficients from adjusted models describing association between SDQ total difficulties scores at age 6 (Parent- and Teacher-rated) and student-teacher relationship quality trajectories (Stable High versus Moderate/Declining), n = 460. [file 40359_2014_27_MOESM1_ESM.docx]

**Additional file 1**

Table 1A.

*Regression Coefficients Describing Association Between Parent-rated SDQ Total Difficulties Scores at Age 6 and Student-Teacher Relationship Quality Trajectories (Stable High versus Moderate/Declining) and Confounding Variables, n = 460*

|  | *Model 2* ^a^ | | | *Model 3* ^b^ | | |
| --- | --- | --- | --- | --- | --- | --- |
|  | *B* | *95% CI* | *p* | *B* | *95% CI* | *p* |
| *Student-Teacher Relationship Quality Trajectories* | | |  |  |  |  |
| Moderate/declining | ref | - | - | ref | - | - |
| Stable High | -3.69 | -5.32 – -2.07 | <.001 | -2.83 | -4.11 – -1.54 | <.001 |
| *Child Preschool Characteristics* |  |  |  |  |  |  |
| Gender (ref male) | -0.45 | -1.41 – 0.51 | .36 | 0.05 | -0.70 – 0.81 | .89 |
| Age in months | 0.06 | -0.06 – 0.18 | .34 | 0.01 | -0.08 – 0.11 | .79 |
| SDQ total difficulties score | - | - | - | 0.72 | 0.64 – 0.81 | <.001 |
| *Family Characteristics During Preschool* | |  |  |  |  |  |
| Parental Warmth | -0.17 | -0.29 – -0.06 | .003 | 0.03 | -0.07 – 0.12 | .58 |
| Parental Distress (GHQ) | 0.38 | 0.18 – 0.58 | <.001 | 0.26 | 0.10 – 0.42 | .001 |
| Single parent (ref no) | 0.89 | -0.71 – 2.50 | .27 | 0.46 | -0.80 – 1.73 | .47 |
| Receiving govt benefit (ref no) | -0.03 | -1.19 – 1.12 | .95 | 0.20 | -0.71 – 1.12 | .66 |
| *%Unemployment (both parents)* |  |  |  |  |  |  |
| Both full time employed | ref | - | - | ref | - | - |
| One full time, one part time | 0.01 | -2.01 – 2.02 | .99 | 0.24 | -1.35 – 1.83 | .76 |
| One full time, or both part time | 0.49 | -1.51 – 2.50 | .63 | 0.65 | -0.93 – 2.23 | .42 |
| One part time, one unemployed | -0.39 | -3.05 – 2.26 | .77 | 0.03 | -2.06 – 2.12 | .98 |
| Both unemployed | -0.18 | -2.81 – 2.45 | .89 | -0.41 | -2.49 – 1.66 | .69 |
| *Mother’s Education* |  |  |  |  |  |  |
| Less than Year 12 | ref | - | - | ref | - | - |
| Year 12 | -1.54 | -2.96 – -0.12 | .03 | -0.87 | -1.99 – 0.26 | .13 |
| TAFE/trade | -0.89 | -2.20 – 0.41 | .18 | -0.65 | -1.68 – 0.38 | .21 |
| Tertiary | -2.79 | -4.35 – -1.23 | .001 | -1.13 | -2.38 – 0.12 | .08 |
| *Father’s Education* |  |  |  |  |  |  |
| Less than Year 12 | ref | - | - | ref | - | - |
| Year 12 | 0.58 | -0.91 – 2.08 | .44 | 0.12 | -1.06 – 1.29 | .84 |
| TAFE/trade | 0.26 | -0.93 – 1.46 | .67 | 0.21 | -0.74 – 1.15 | .67 |
| Tertiary | -0.87 | -2.67 – 0.93 | .34 | -0.49 | -1.91 – 0.93 | .50 |

*Note.* Ref = reference category.

^a^ Model 2 adjusted for: child age at preschool, child gender, Parent distress, Parental warmth, maternal and paternal education and employment, receiving welfare benefit, and single parent family status. ^b^ Model 3 adjusted for: child age at preschool, child gender, Parent distress, Parental warmth, maternal and paternal education and employment, receiving welfare benefit, and single parent family status, and the corresponding informant-specific SDQ Total difficulties score at age 4 (parent-rated and teacher-rated, respectively).

Table 2A.

*Regression Coefficients Describing Association Between Teacher-rated SDQ Total Difficulties Scores at Age 6 and Student-Teacher Relationship Quality Trajectories (Stable High versus Moderate/Declining) and Confounding Variables, n = 460*

|  | *Model 2* ^a^ | | | *Model 3* ^b^ | | |
| --- | --- | --- | --- | --- | --- | --- |
|  | *B* | *95% CI* | *p* | *B* | *95% CI* | *p* |
| *Student-Teacher Relationship Quality Trajectories* | | |  |  |  |  |
| Moderate/declining | ref | - | - | ref | - | - |
| Stable High | -10.15 | -11.79 – -8.51 | <.001 | -7.98 | -9.69 – -6.28 | <.001 |
| *Child Preschool Characteristics* |  |  |  |  |  |  |
| Gender (ref male) | -2.07 | -3.04 – -1.10 | <.001 | -1.54 | -2.48 – -0.59 | .001 |
| Age | 0.11 | -0.01 – 0.24 | .08 | 0.11 | -0.01 – 0.23 | .07 |
| SDQ total difficulties score | - | - | - | 0.33 | 0.23 – 0.43 | <.001 |
| *Family Characteristics During Preschool* | |  |  |  |  |  |
| Parental Warmth | -0.01 | -0.13 – 0.10 | .82 | -0.04 | -0.15 – 0.08 | .52 |
| Parental Distress (GHQ) | 0.03 | -0.17 – 0.22 | .80 | -0.01 | -0.21 – 0.18 | .88 |
| Single parent (ref no) | 1.37 | -0.26 – 2.99 | .09 | 1.25 | -0.30 – 2.81 | .11 |
| Receiving govt benefit (ref no) | 0.18 | -0.99 – 1.35 | .77 | 0.19 | -0.93 – 1.31 | .74 |
| *%Unemployment (both parents)* |  |  |  |  |  |  |
| Both full time employed | ref | - | - | ref | - | - |
| One full time, one part time | -0.03 | -2.07 – 2.01 | .98 | 0.17 | -1.78 – 2.13 | .86 |
| One full time, or both part time | -0.61 | -2.63 – 1.42 | .56 | -0.51 | -2.45 – 1.43 | .61 |
| One part time, one unemployed | -0.31 | -2.99 – 2.38 | .82 | -0.49 | -3.06 – 2.07 | .71 |
| Both unemployed | -0.29 | -2.95 – 2.37 | .83 | -0.33 | -2.88 – 2.21 | .79 |
| *Mother’s Education* |  |  |  |  |  |  |
| Less than Year 12 | ref | - | - | ref | - | - |
| Year 12 | -0.98 | -2.42 – 0.46 | .18 | -0.55 | -1.94 – 0.83 | .43 |
| TAFE/trade | -0.96 | -2.82 – 0.36 | .15 | -0.75 | -2.01 – 0.51 | .25 |
| Tertiary | -1.06 | -2.64 – 0.53 | .19 | -0.82 | -2.33 – 0.70 | .29 |
| *Father’s Education* |  |  |  |  |  |  |
| Less than Year 12 | ref | - | - | ref | - | - |
| Year 12 | -0.01 | -1.52 – 1.51 | .99 | -0.13 | -1.58 – 1.32 | .86 |
| TAFE/trade | -0.54 | -1.76 – 0.67 | .38 | -0.67 | -1.83 – 0.49 | .26 |
| Tertiary | -1.10 | -2.92 – 0.72 | .23 | -1.01 | -2.75 – 0.73 | .26 |

*Note.* Ref = reference category.

^a^ Model 2 adjusted for: child age at preschool, child gender, Parent distress, Parental warmth, maternal and paternal education and employment, receiving welfare benefit, and single parent family status. ^b^ Model 3 adjusted for: child age at preschool, child gender, Parent distress, Parental warmth, maternal and paternal education and employment, receiving welfare benefit, and single parent family status, and the corresponding informant-specific SDQ Total difficulties score at age 4 (parent-rated and teacher-rated, respectively).
